# Defining micro-epidemiology for malaria elimination: systematic review and meta-analysis

**DOI:** 10.1186/s12936-017-1792-1

**Published:** 2017-04-20

**Authors:** Melanie Bannister-Tyrrell, Kristien Verdonck, Susanna Hausmann-Muela, Charlotte Gryseels, Joan Muela Ribera, Koen Peeters Grietens

**Affiliations:** 1 Institute of Tropical Medicine, Antwerp, Belgium; 2 Swiss Agency for Development and Cooperation, Bern, Switzerland; 3 MARC-Universitat Rovira i Virgili, Tarragona, Spain

**Keywords:** Micro-epidemiology, Malaria elimination, Hotspot, Fine-scale heterogeneity, Epidemiology methods

## Abstract

**Background:**

Malaria risk can vary markedly between households in the same village, or between villages, but the determinants of this “micro-epidemiological” variation in malaria risk remain poorly understood. This study aimed to identify factors that explain fine-scale variation in malaria risk across settings and improve definitions and methods for malaria micro-epidemiology.

**Methods:**

A systematic review of studies that examined risk factors for variation in malaria infection between individuals, households, clusters, hotspots, or villages in any malaria-endemic setting was conducted. Four databases were searched for studies published up until 6th October 2015. Crude and adjusted effect estimates for risk factors for malaria infection were combined in random effects meta-analyses. Bias was assessed using the Newcastle–Ottawa Quality Assessment Scale.

**Results:**

From 743 retrieved records, 51 studies were selected, representing populations comprising over 160,000 individuals in 21 countries, in high- and low-endemicity settings. Sixty-five risk factors were identified and meta-analyses were conducted for 11 risk factors. Most studies focused on environmental factors, especially increasing distance from a breeding site (OR 0.89, 95% CI 0.86–0.92, 10 studies). Individual bed net use was protective (OR 0.63, 95% CI 0.52–0.77, 12 studies), but not household bed net ownership. Increasing household size (OR 1.08, 95% CI 1.01–1.15, 4 studies) and household crowding (OR 1.79, 95% CI 1.48–2.16, 4 studies) were associated with malaria infection. Health seeking behaviour, medical history and genetic traits were less frequently studied. Only six studies examined whether individual-level risk factors explained differences in malaria risk at village or hotspot level, and five studies reported different risk factors at different levels of analysis. The risk of bias varied from low to high in individual studies. Insufficient reporting and comparability of measurements limited the number of meta-analyses conducted.

**Conclusions:**

Several variables associated with individual-level malaria infection were identified, but there was limited evidence that these factors explain variation in malaria risk at village or hotspot level. Social, population and other factors may confound estimates of environmental risk factors, yet these variables are not included in many studies. A structured framework of malaria risk factors is proposed to improve study design and quality of evidence in future micro-epidemiological studies.

**Electronic supplementary material:**

The online version of this article (doi:10.1186/s12936-017-1792-1) contains supplementary material, which is available to authorized users.

## Background

Heterogeneity in malaria risk at fine spatial scales is well recognized and factors that may contribute to this fine-scale heterogeneity were described nearly 30 years ago, and include genetic, social and environmental factors affecting exposure and response to infection [1]. As malaria control efforts progress towards elimination, it is increasingly important to understand the factors that influence the persistence of malaria transmission at fine spatial scales. Malaria transmission may persist in ‘hotspots’ or ‘hotpops’ despite application of standard control measures, even when malaria incidence in the surrounding region decreases [2]. Coarse-scale data on determinants of malaria incidence (e.g. collected at district, regional or national level) may not be readily interpolated to predict transmission in these contexts of residual persistent transmission, as it may mask fine-scale heterogeneity and the role of local contextual factors. At this scale household construction, local mobility patterns, land use, health-seeking behaviour and other local contextual factors may be important determinants of heterogeneity. Greater insights into the causes of fine-scale heterogeneity in malaria transmission may improve the application of interventions to target hotspots [3].

Several studies have recently described and analysed micro-epidemiological variation in malaria risk at different endemicity levels [2, 4–7], coinciding with an increased interest in operationalizing novel tools for malaria risk stratification [8]. Malaria risk stratification is recommended by the World Health Organization [9], but has been criticized for being too complex to be useful for implementation while still too general to adequately describe local malaria patterns [8, 10]. Since the ‘micro-epidemiology’ of malaria was first described, there has been relatively little discussion in the literature about the impact of micro-epidemiological risk factors in explaining variation in malaria risk in different transmission contexts, and the generalizability of micro-epidemiological studies to other settings is unclear. Given the emphasis on tailoring malaria interventions to local contexts and improving risk stratification as part of the global technical strategy to control and eliminate malaria [9], there is a clear need to define the scope, theory and methods for micro-epidemiological studies of malaria.

The aims of this study are to identify factors that explain micro-epidemiological variation in risk, and to contribute to the development of theory and methods in the field of malaria micro-epidemiology.

## Methods

### Protocol registration

A protocol for this review was prepared but not registered because it does not concern an intervention and at present, systematic reviews of risk factors in observational studies are not eligible for registration with the PROSPERO, Cochrane or Campbell systematic review registries. This review is reported according to PRISMA guidelines [11].

### Working definition of micro-epidemiology

A working definition of ‘micro-epidemiology’ was developed based on a preliminary review of the literature, guiding the selection of studies. ‘Micro-epidemiology’ was considered to encompass studies assessing variation in measures of *Plasmodium* infection frequency between households or other sub-village groupings within villages, or between neighbouring villages or other similar socio-spatial aggregations such as urban neighbourhoods, agricultural settlements and health centre catchment areas.

### Study design and setting

Observational studies in any setting where human *Plasmodium* transmission occurs were included, except studies of sporadic imported malaria cases, or limited outbreaks of autochthonous malaria transmission following an imported case in settings that are otherwise malaria-free.

### Outcome of interest

The primary outcome was defined as current or recent *Plasmodium* infection in a person, which is parasitologically or serologically confirmed. This outcome definition differs somewhat from the revised standard World Health Organization definition of malaria case, which is based on current infection only [12], as studies suggest that serology outcomes are a more stable marker of malaria risk than *Plasmodium* infection prevalence in cross-sectional studies, particularly in low-endemicity settings [2].

### Independent variables of interest

No restriction was applied to the types of risk factors included in studies, as the aim was to canvas the scope of risk factors that potentially explain variation in *Plasmodium* infection at micro-spatial scales. Studies were excluded if they did not present any risk factor analyses for *Plasmodium* infection.

### Information sources and search

The primary information source for this study was the PubMed database, and ISI Web of Knowledge, LILACS and Google Scholar were used as secondary databases. The search strategy below was used to retrieve titles and abstracts of potentially relevant studies in PubMed. The search strategy was constructed using the PubMed advanced search builder and run on 6th October 2015, without date restriction. An additional search was run excluding MeSH terms for the years 2014 and 2015 to allow for retrieval of articles that have not yet been indexed. The review was restricted to studies published in English. In all databases, additional searches for ‘malaria small area studies’ and ‘malaria local variation’ did not yield additional relevant papers beyond those already identified. Additionally, reference lists of key articles were checked for additional studies.

((malaria or Plasmodium or Anopheles [title/abstract]) and (‘micro-epidemiology’ or ‘microepidemiology’ or ‘micro epidemiology’ or ‘hotspot’ or ‘heterogeneity’ or ‘cluster*’ or ‘spatial cluster*’) and (‘malaria/epidemiology’ or ‘malaria/ethnology’ or ‘malaria/statistics and numerical data’ [mesh major topic]))

### Study selection

Two authors independently screened titles and abstracts, selected articles for full-text review, and made the final article selection. The final list of articles selected were compared, and in case of uncertainty or disagreement about whether a record was eligible for inclusion in the review, it was discussed amongst the two reviewers until consensus was reached.

### Data collection process

All retrieved citations were exported into an Endnote X7 library. Titles, abstracts and the selected full-text articles were reviewed in Endnote, and data were extracted into a piloted, pre-specified table in Microsoft Excel for studies that met the inclusion criteria. The following items were collected: study population and location; malaria species; vector(s); study design; sample size (individuals, households, villages); time period of study; spatial scale of study; malaria prevalence/incidence; malaria case detection method (passive case detection, active case detection, population-based screening); malaria diagnostic; risk factors (see below for classification scheme); and, analytical methods. From the results of each study, risk factors reported to be significantly associated with malaria risk as defined in each study (typically p < 0.05) were collected, including effect estimates and 95% confidence intervals where presented. For descriptive and qualitative studies, significance was broadly defined as the authors attributing observed heterogeneity in malaria infection to a risk factor based on presented data, such as site maps (e.g., for attributing variation between villages to proximity to forest), frequency tables or qualitative findings, but these studies were not incorporated into meta-analyses.

Malaria risk factors were initially extracted as reported, and then grouped into variables. For example, reported items such as used a bed net last night, owns a bed net, long-lasting insecticidal net (LLIN) or insecticide-treated net (ITN) use were grouped as ‘bed net use/ownership’, with distinctions made between whether a variable was measured at individual, household or other level. From these initial groupings, risk factors were further classified using the following classification scheme that was developed a priori and refined upon record review and extraction.

#### Demographic

Personal characteristics such age, gender, ethnicity, socio-economic status, migrant status, which influence malaria risk by modifying or acting through factors described below.

#### Social

all variables describing social patterns and behaviours that may directly or indirectly modify exposure to biting vectors, such as bed net use and outdoor activities.

#### Environmental

Variables measuring relative exposure to biting mosquitoes related to physical or landscape features such as proximity to vector breeding sites, landscape features and weather and climate conditions.

#### Medical history and genetic traits

Human host and genetic factors related to development of parasitaemia and clinical disease once exposed to an infectious bite, such as immune status, co-infections and genetic traits.

#### *Plasmodium* and human population

Variables measuring exposure to local *Plasmodium* populations, including household malaria cases or residence in hotspot, as well as prevalence of drug-resistant strains. Household and village population size were also included because here they affect exposure to *Plasmodium* populations as a function of the number and density of available human hosts.

#### Health seeking behaviour and access to care

Variables related to seeking testing and treatment for malaria, including knowledge and perceptions of malaria illness, access to and availability of malaria control programmes, provider and treatment preferences.

### Risk of bias in individual studies

Risk of bias in individual studies was assessed using the Newcastle–Ottawa scale for assessing quality of nonrandomized studies in meta-analyses [13]. The quality of studies is assessed across three domains, including selection of the study groups, the comparability of the groups and the ascertainment of either the exposure or outcome of interest, for cohort and case–control studies, respectively. Cross-sectional studies were assessed using the same quality criteria as the Newcastle–Ottawa scale for case–control studies. Within each domain, quality is assessed using a star-based scoring system, with a maximum of one star per item in the ‘selection’ and ‘exposure/outcome’ domains, and a maximum of two stars in the ‘comparability between groups’ domain. Studies were awarded one star for comparability if they adjusted for at least one factor(s) from the categories in the classification scheme, and two stars if they adjusted for factors from two or more categories. Each study can be awarded a maximum of nine stars across the three domains combined.

### Synthesis of results

#### Descriptive synthesis

Included studies were described in terms of study setting, study design, spatial area, endemicity, and level of analysis (individual, household, village/cluster). Frequency tables of risk factors were produced, contrasting frequency with which a risk factor was studied to the frequency that significant associations (typically p < 0.05) for each factor were reported.

#### Quantitative synthesis

Results from individual studies were combined in meta-analyses to estimate the magnitude of effect sizes and heterogeneity of effects across studies. Several methods were used to generate effect estimates that were not presented in the required form for meta-analysis in the individual publications, which are fully described in the Additional file 1. Pooled estimates are presented in-text where calculated, otherwise the total number of studies assessing each variable and the number of significant associations reported are described.

Heterogeneity by relative risk measure (odds ratio, rate ratio, risk ratio), *Plasmodium* or vector species, study setting, and various other sources of heterogeneity were explored qualitatively but there were too few studies per variable to stratify on study design or risk measure. All effect estimates were assumed to estimate the odds ratio, as this was the most commonly calculated measure. Heterogeneity between study estimates included in meta-analysis was assessed using the I^2^ statistic.

To synthesize the descriptive and quantitative findings, a conceptual framework was developed for the relationships between risk factors for which there is evidence of association with malaria infection risk in different settings.

### Risk of bias across studies

Risk of bias across studies in meta-analyses was considered high because the search strategy was systematic with respect to study design only, not individual variables, and furthermore, many studies did not present effect estimates (including unadjusted or adjusted) for variables reported to be non-significant. Statistical tests of significance for pooled effect estimates are not presented because the high risk of bias limits meaningful interpretation of p values, and the effect estimates and confidence intervals should be considered indicative rather than conclusive.

## Results

### Study selection

**Fig. 1:**
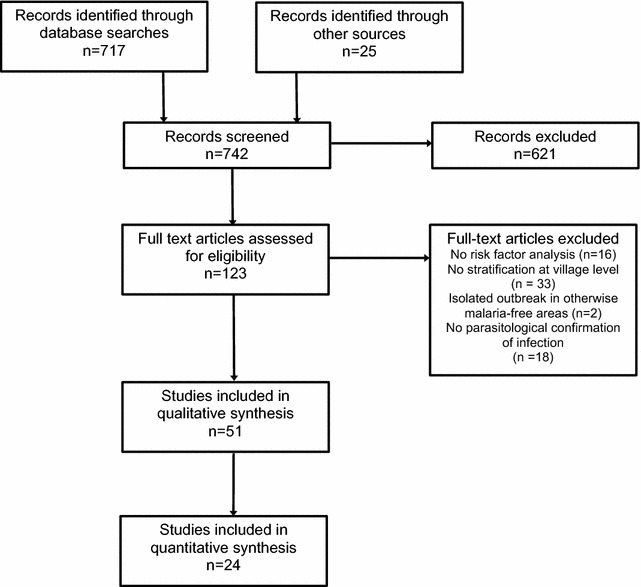
Flowchart for selection of articles in systematic review

Some 717 records were retrieved across the database searches and 25 additional titles were retrieved from article reference lists. 121 articles were selected for full-text review, including five that were identified from reference list screening (Fig. 1). In total, 51 articles published between 1986 and 2015 and based on data collected in 45 locations comprising a total study population approximating 160,000 individuals, were included in the review.

### Study characteristics (Table 1)

#### Study settings

Micro-epidemiological studies of malaria transmission have been conducted on all continents, in high and low endemicity settings. Most studies (n = 44) were conducted in rural settings including coastal, forest/forest fringe, highland, and large agricultural settlement sites. There were six studies conducted in urban or peri-urban settings and one study that contrasted a peri-urban to a nearby rural setting [5]. There were 29 articles that described sub-Saharan African study sites focusing mainly on falciparum malaria, including rural and urban study settings. In the 12 Asian study sites there was greater vector diversity than in African sites and two to four *Plasmodium* species were present. Ten studies in Latin America mostly focused on vivax malaria (Table 1).

**Table 1:** Characteristics of studies included in systematic review of micro-epidemiological studies of malaria

Study	Study setting	Malaria species	Dominant vector(s)	Study design	Number of participants	Number of clusters	Infections (n, case-only studies), prevalence(%) or incidence	Spatial scale	Duration	Detection (diagnostic)	Units of analysis	Quality (max 9 stars)^a^
Alemu et al. [20]	Dabat district, Ethiopian Highlands	*P. falciparum, P. vivax*	Not specified	Case control	1644	4 health facilities	645	1187.9 km^2^	10 months	PCD (RDT, LM)	Individual, cluster (hotspot)	★★★★★★
Atieli et al. [23]	Western Kenyan Highlands	*P. falciparum*	*An gambiae*	Cross sectional	480 households	4 villages	0.7–20.6%	36 km^2^	1 month	Screening (LM)	Cluster (valley location)	★★★★★★
Audibert et al. [14]	Logone Valley, Cameroon	*P. falciparum*	Not specified	Cross sectional (pre-post)	716–1078	3 villages	7.1–30.1%	350 km^2^	5 years	Screening (LM)	Cluster (village)	★★★★★★
Baragatti et al. [22]	Ouagadougou, Burkina Faso	*P. falciparum*	*An. gambiae*	Cross-sectional	3354	8 villages	22%	144 km^2^	1 year	Screening (LM)	Individual, cluster (urban areas)	★★★★★★★★
Barrera et al. [15]	Sucre State, Venezuela	*P. vivax*	*An. aquasalis curry*	Cohort	8836	35 localities	Not reported	51 km^2^	12 years	PCD (LM)	Cluster (village)	★★★★★★★
Barros and Honario [41]	Rorainopolis settlement, Roraima Province, Brazil	*P. vivax*	*An. darlingi*	Cohort	75 households	1 settlement	186	18.8 km road	2.5 years	PCD (LM)	Household	★★★★★
Basurko et al. [42]	Cacao village, French Guiana	*P. vivax*	*An. darlingi*	Cohort	839	1 village	359	1 km^2^	5 years	PCD (LM)	Individual	★★★★
Bejon et al. [43]	Kilifi district, Kenya	*P. falciparum*	*An. gambiae*	Cohort	1708	3 villages	0.49–0.82 episodes/child/year	450 km^2^	11 years	Screening, ACD (LM)	Household (homestead)	★★★★★★★
Bousema et al. [2]	Korogwe district, Tanga Region, Tanzania	*P. falciparum*	*An. gambiae*	Cohort	1276	15 villages	399	400 km^2^	4 years	PCD (RDT)	Cluster (hotspot)	★★★★★★
Brooker et al. [44]	Nandi district, Kenya	*P. falciparum*	*An. gambiae*	Case control	284	3 villages	129	144 km^2^	10 weeks	ACD (LM)	Individual	★★★★★★★
Camargo et al. [45]	Urupa Farm, Rondonia, Brazil	*P. falciparum, P. vivax*	*An. darlingi*	Cohort	Average 170, varied seasonally	1 settlement	171	230 km^2^	2 years	Screening, PCD (LM)	Individual	★★★★★★
Cattani et al. [16]	Villages around Madang, Papua New Guinea	*P. falciparum*	Not specified	Repeated cross sectional	~16,500 in area	6 villages	41–89%	900 km^2^	2 years	Screening (LM)	Cluster (village)	★★★★★★★
Clark et al. [28]	Mulago III parish, Kampala, Uganda	*P. falciparum*	*An. gambiae*	Cohort	558	1 village	0.77 episodes/person/year	1 km^2^	2 years	PCD (LM)	Individual	★★★★★★★★★
Coulibaly et al. [46]	Bandiagara, Mali	*P. falciparum*	*An. gambiae*	Cohort	300	1 village	296	4 km^2^	1 year	Screening, PCD (LM)	Individual	★★★★★
da Silva-Nunes et al. [24]	Pedro Peixoto settlement, Acre, Brazil	*P. falciparum, P. vivax*	*An. darlingi*	Cohort	509	1 settlement	15.5% by PCR, 5.2% by LM	15 km^2^	2 years	Screening, ACD, PCD (LM, PCR)	Individual	★★★★★★★
de Barros et al. [41]	Rorainopolis settlement, Roraima Province, Brazil	*P. vivax*	*An. darlingi*	Cohort	333	1 settlement	31% ≥1 episode	18.8 km road	2.5 years	PCD (LM)	Household	★★★★★★★
Ernst et al. [47]	Kipsamoite, North Nandi district, Kenya	*P. falciparum*	*An. gambiae, An. funestus*	Cohort	3700	7 villages	41–127 episodes/1000 persons/year	16 km^2^	4 years	PCD (LM)	Individual, household	★★★★★★★
Florey et al. [26]	Kingwede village, Kenya	*P. falciparum, P. malariae, P. ovale*	Not specified	Cross-sectional	561	1 village	50.40%	10 km^2^	3 months	Screening (PCR)	Individual	★★★★★★★★
Gamage-Mendis et al. [48]	Kataragama area, Sri Lanka	*P. vivax, P. falciparum*	*An. subpictus, An. culicifacies*	Cohort	3023	6 villages	25.80%	8 km^2^	17 months	PCD (LM)	Individual, household	★★★★★
Gaudart et al. [49]	Bancoumana village, Mali	*P. falciparum, P. malariae, P. ovale*	Not specified	Cohort	1101–1491	1 village	47%	2.5 km^2^	5 years	Screening (LM)	Individual	★★★★★★★★★
Ghebreyesus et al. [50]	Tigray Region, Ethiopia	*P. vivax, P. falciparum*	*An. arabiensis*	Cohort	2114	6 villages	Not reported	Not reported	1 year	Screening (LM)	Individual	★★★★★★★★
Grange et al. [27]	Dielmo village, Senegal	*P. falciparum*	Not specified	Cohort	828	1 village	898 gametocyte-positives in 297 individuals	Not reported	19 years	Screening, ACD (LM)	Individual	★★★★★★★
Grillet, Barrera et al. [17]	Caijigal Municipality, Sucre State, Venezuela	*P. vivax*	*An. aquasalis curry*	Cohort	24,345	29 villages	10-44 cases/1000 persons/year	332.5 km^2^	7 years	ACD, PCD (LM)	Cluster (village)	★★★★★★★★
Grillet, Jordan, et al. [18]	Caijigal Municipality, Sucre State, Venezuela	*P. vivax*	*An. aquasalis curry*	Cohort	24,788	29 villages	10-44 cases/1000 persons/year	332.5 km^2^	7 years	ACD, PCD (LM)	Cluster (village)	★★★★★
Gunawardena, et al. [51]	Kataragama area, Sri Lanka	*P. vivax, P. falciparum*	*An. culicifacies*	Cohort	1744	8 villages	0.91 cases/person/18 months	26 km^2^	18 months	Screening, PCD (LM)	Household	★★★★★
Haque, Glass et al. [52]	Gilachari Union, Rangamati district, Chittagong Hill Tracts	*P. falciparum*	*An. baimai, An. minimus, An. annularis*	Cohort	7922	54 villages	6.30%	113.83 km^2^	2 years	PCD (RDT, LM)	Individual, household	★★★★★★★
Haque, Magalhaes et al. [29]	Rajasthali sub-district, Chittagong Hill districts, Bangladesh	*P. falciparum, P. vivax*	*An. baimai, An. minimus, An. annularis*	Cross-sectional	1400	109 villages	11.50%	145 km^2^	<1 year	Screening (RDT)	Individual	★★★★★★★★
Haque, Sunahara et al. [7]	Rajasthali sub-district, Chittagong Hill districts, Bangladesh	*P. falciparum, P. vivax*	Not specified	Cross sectional	1400	109 villages	11.50%	250 km^2^	1 month	Screening (RDT)	Individual	★★★★★★★★
Kreuels et al. [19]	Afigya-Sekyere district, Ashanti region, Ghana	*P. falciparum*	*An. gambiae, An. funestus*	Cohort	535	9 villages	67% individuals ≥1 episode	200 km^2^	21 months	PCD (LM)	Individual, cluster (village)	★★★★★★
Loha et al. [37]	Chano Mille kebele, Ethiopia	*P. falciparum, P. vivax*	*Not specified*	Cohort	8121	1 village	45.1 episodes/1000 persons/year	2.4 km^2^	2 years	ACD, PCD (RDT, LM)	Individual, household	★★★★★★★
Luxemburger et al. [53]	Karen refugee camp, Thailand	*P. falciparum, P. vivax*	*An. minimus, An. maculatus*	Cohort	735	1 refugee camp	4%	2 km^2^	1 year	ACD, PCD (LM)	Individual	★★★★★★★
Midega et al. [54]	Kilifi district, Kenya	*P. falciparum*	*An. gambiae*	Cohort	642	338 homesteads	14% by PCR, 0.7 episodes/child/year	40 km^2^	1 year	Screening, ACD (LM)	Individual	★★★★★★★★
Mosha et al. [6]	Misungwi district, Tanzania	*P. falciparum*	*An. gambiae*	Repeated cross sectional	3426	4 villages	49%	Not reported	4 months	Screening (PCR, serology)	Individual	★★★★★★★★
Mosha et al. [55]	Misungwi district, Tanzania	*P. falciparum*	*An. gambiae*	Cross sectional	3057	4 villages	35.20%	Not reported	4 months	Screening (PCR)	Household	★★★★★★★
Murhandarwati et al. [38]	Kokap subdistrict, Kulon Progo, Indonesia	*P. vivax, P. falciparum*	*An. maculatus, An. balabacensis, An. vagus*	Mixed methods	42,264	5 villages	0.50%	90 km^2^	1 year	ACD, PCD (LM)	Individual	★★
Ndiath et al. [56]	Keur Soce DHS site, Senegal	*P. falciparum*	Not specified	Cross sectional	1614	74 villages	12%	312 km^2^	1 month	ACD (RDT)	Individual	★★★★★★★★
Nixon et al. [57]	Wainyapu village, Sumba, Indonesia	*P. falciparum, P. vivax, P malariae*	*An. sundaicus, An. subpictus, An. vagus*	Cross sectional	960	1 village	25%	22 km^2^	4 months	Screening (LM)	Individual	★★★★★★★★
Olotu et al. [58]	Kilifi district, Kenya	*P. falciparum*	*An. gambiae*	Cohort	2425	3 villages	1.4 episodes/person/year	450 km^2^	12 years	ACD, PCD (LM)	Individual	★★★★★★
Parker et al. [59]	Thailand/Myanmar border	*P. vivax, P. falciparum, P. malariae*	Not specified	Cohort	Average 494	1 village	75	0.16 km^2^	10 months	Screening (LM, PCR)	Individual	★★★★★★★★
Peterson, Borrell et al. [60]	Kebele 11, Adama City, Ethiopia	*P. vivax, P. falciparum*	*An. arabiensis*	Cohort	1367	1 kebele	9%	1.8 km^2^	4 months	PCD (LM)	Individual, household	★★★★★★★
Prakash, Mohapatra, 2000 [21]	Nedeluajan village, Jorhat district, Assam, India	*P. falciparum, P. vivax*	*An. dirus, An. minimus*	Cross sectional	701	3 sub-village clusters, 1 village	16%	1 km^2^	1 month	Screening (LM)	Individual, cluster (sub-village)	★★★★★★
Pullan, Bukirwa et al. [61]	Mulanda sub-county, Tororo district, Uganda	*P. falciparum*	*Anophelees gambiae, An. funestus*	Cross sectional	1844	4 villages	39%	7.5 km^2^	4 months	Screening (RDT)	Individual	★★★★★★★★
Pullan, Kabatereine et al. [25]	Mulanda sub-county, Tororo district, Uganda	*P. falciparum, P. malariae*	*Anophelees gambiae, An. funestus*	Cross sectional	1770	14 clusters, 4 villages	39%	7.5 km^2^	4 months	Screening (RDT)	Individual	★★★★★★★★
Rosas-Aguirre, Ponce et al. [62]	Bellavista district, Sullana province, Peru	*P. vivax*	*An. albimanus*	Cross sectional, case control	4650	3 neighbourhoods	13% ≥1 episode	3.1 km^2^	2 years	PCD (LM, PCR)	Household	★★★★★
Rosas-Aguirre, Speybroeck et al. [63]	San Juan, Loreto region, Peru	*P. vivax, P. falciparum*	*An. darlingi*	Cross sectional	651	3 communities	3% by MS, 11% by PCR	48 km road	1 month	Screening (LM, PCR, serology)	Individual	★★★★★★★★
Rulisa et al. [64]	Ruhuha Sector, Bugesera district, Rwanda	*P. falciparum*	Not specified	Cross sectional	769	1 sector	23% of self-reported fever cases	54 km^2^	7 months	ACD, PCD (RDT)	Individual, household	★★★★★★
Sissoko et al. [5]	Sotuba and Kolle villages, Mali	*P. falciparum*	*An. gambiae, An. funestus*	Cross sectional	588	2 villages	8–35%	2 km^2^	6 months	Screening, PCD (LM)	Individual, cluster (hotspot)	★★★★★★★
Trape et al. [65]	Pikine Ancien district, Dakar, Senegal	*P. falciparum, Plasmodium. malariae*	*An. arabiensis*	Repeated cross sectional	2465	1 urban sub-district	4%	910 m transect	9 months	Screening (LM)	Individual	★★★★★★
van der Hoek et al. [66]	Mahameegaswewa village, Anuradhapura district, Sri Lanka	*P. vivax, P. falciparum*	*An. culicifacies*	Cohort	280	1 village	1.5 episodes/person/year	1 village	11 months	ACD (LM)	Individual	★★★★★★★★
Winskill et al. [67]	Muheza district, Tanga region, Tanzania	*P. falciparum*	*An. gambiae sl*	Cross sectional	1438	21 hamlets, 5 villages	15%	300 km^2^	Not specified	Screening (LM)	Individual	★★★★★★★★
Ye et al. [4]	Kossi province, Burkina Faso	*P. falciparum*	Not specified	Cohort	867	3 villages, 1 town	787.6 episodes/person/year	19–44 km between villages	1 year	ACD (LM)	Individual, cluster (village)	★★★★★★★★

*ACD* active case detection, *LM* light microscopy, *RDT* rapid diagnostic test, *PCR* polymerase chain reaction

^a^Quality assessed using the Newcastle–Ottawa Quality Assessment Scale for observational studies

#### Spatial scales of micro-epidemiological studies of malaria

The spatial scale of study sites ranged from <1 to 1188 km^2^, with a median of 38 km^2^. Many studies were conducted within one or a few neighbouring villages or sub-village clusters, with the largest study conducted in 109 villages in a 145 km^2^ district.

#### Units of analysis

There were 12 studies that examined risk factors for aggregated malaria infections or risk of infection, including six studies that investigated risk factors for malaria risk at village level [4, 14–19], three studies that described risk factors for residing in a malaria ‘hotspot’ [2, 5, 20], one that analysed sub-village geographical clusters [21], one that compared urban areas [22], and one study that compared malaria risk by topography type [23]. Household-level analyses were conducted in 12 studies, with malaria risk factors in the remaining 26 studies analysed at individual level and data showing spatial clustering of malaria infections presented separately.

### Risk factors for malaria in micro-epidemiological studies

#### Demographic factors

Most but not all studies included basic demographic variables such as age, gender and a measure of income or wealth, and often occupation and education level (Table 2). Age was associated with individual malaria risk in most studies (25/36), whereas gender and wealth status were not (5/30 and 6/18 respectively). In four of five studies in which gender was associated with malaria risk, adult males working away from the home in outdoor occupations represented the highest risk group. Ethnicity was associated with malaria risk in four of five studies, although in all cases authors report that ethnicity is collinear with village location. Migrants and people lacking citizenship were reported to have higher risk of malaria in three of five studies.

**Table 2:** Variables included as malaria risk factors in 51 micro-epidemiological studies included in the systematic review

Variables included as risk factors for malaria in 51 studies	Studies including this variable	Significant association reported
*Demographic factors*		
Age	[4, 6, 7, 15, 16, 20–22, 24–29, 37, 42, 44, 45, 47, 49, 50, 52, 53, 55–67]	[4, 6, 7, 15, 16, 20–22, 25–27, 29, 37, 42, 45, 52, 53, 55–57, 63–67]
Gender	[4, 6, 7, 15, 19–22, 24–26, 28, 29, 37, 42, 44, 45, 50, 52, 53, 56, 57, 59–64, 66, 67]	[7, 20, 42, 45, 64]
Ethnicity	[4, 7, 19, 29, 38]	[4, 19, 29, 38]
Income/wealth status	[2, 6, 16, 19, 22, 24–26, 28, 37, 44, 50, 56, 61–63, 66, 67]	[2, 19, 22, 26, 37, 56]
Occupation	[7, 19, 24, 29, 45, 62]	[19, 45]
Educational level	[6, 7, 19, 22, 24–26, 29, 52, 60, 62]	[6, 19, 22]
Migrant status	[24, 38, 42, 59]	[38, 42]
Citizenship status	[59]	[59]
Marital status	[60]	
*Social factors*		
Number of sleeping rooms in house	[50, 67]	[50]
Number of occupants per sleeping room	[2, 60, 68]	
Household dependency ratio	[59, 60]	[60]
Presence of household guests	[60]	
Individual bed net ownership/use	[2, 4, 6, 7, 19, 20, 22, 24–26, 28, 29, 37, 44, 53, 56, 60, 61, 66–68]	[2, 5, 6, 19, 25, 28, 56, 61, 66, 67]
Household bed net ownership/use	[2, 5, 7, 25, 29, 52, 57, 60–62, 64]	[7, 25, 52, 60, 64]
Use of coils, repellent, fumigants to deter vectors	[2, 44, 66, 68]	[66]
Recent travel away from primary residence	[20, 22, 24, 53, 60, 68]	[20, 53, 60]
Outdoor occupation	[24, 38, 45, 60, 63]	[24, 45, 63]
Household member in outdoor occupation	[24, 60, 62]	[24, 60]
Evening outdoor activities	[26, 38, 68]	[26]
Dawn activities	[38]	
Water contact behaviours (e.g. fishing, bathing)	[24, 26]	[24]
*Environmental factors*		
Housing construction quality	[6, 24, 28, 38, 48, 51, 57, 61, 66]	[6, 48, 51]
House roofing material	[2, 44, 47, 49, 50, 63, 64, 68]	[2, 47, 50, 68]
House wall material	[2, 24, 47, 52, 62, 64, 67, 68]	[2, 52, 64, 68]
House floor material	[5, 7, 25, 29, 56, 62]	[7, 25, 56]
Presence/type of eaves	[2, 5, 44, 50, 67]	[5, 50]
Presence/type of windows	[2, 5, 19, 24, 50, 56, 60, 67]	[5, 19, 50]
Separate kitchen	[24, 50]	[50]
House size (spatial area)	[67]	
Household water source	[28, 50, 62, 64]	[62]
House treated with indoor residual spraying	[6, 20, 64]	
Household Solid and liquid waste disposal	[24, 25, 60, 62]	
Household surroundings (garden, litter, tidiness)	[20, 60, 64]	[60]
Proximity to vector breeding site	[2, 4–6, 15, 17, 37, 41, 54, 57, 60, 68]	[5, 6, 15, 17, 37, 41, 54, 55, 57, 60, 68]
Proximity to water body (e.g. pond, lake, swamp, stream)	[22, 26, 28, 41, 46, 47, 51, 52, 65, 66, 68]	[28, 46, 47, 51, 65, 66]
Proximity to man-made water storage and management (well, drain, piped water, brickworks)	[4, 22, 46, 47, 49, 57, 62]	[22, 46, 49, 62]
Proximity to forest	[19, 21, 41, 44, 47, 48, 51]	[19, 21, 41, 47]
Local forest density	[7, 29]	[7, 29]
Proximity to agriculture (e.g. rice irrigation, tea plantation)	[4, 14, 25, 44, 50, 61]	[4, 25, 50, 61]
Vector breeding site density	[2, 15, 17, 23, 54]	[2]
Direction of nearest vector breeding site	[54]	[54]
Number of households on path to breeding site	[37, 54]	[37]
Adult vector density	[5, 23, 48]	
Exposure to infectious biting mosquitoes	[2]	[2]
Domestic animals kept in/near house	[2, 4, 6, 14, 24, 38, 44, 50, 56, 60, 62, 64, 66, 67]	[50, 56, 60, 62]
House location	[22, 24, 42, 45]	[24]
Proximity to main road	[17, 47, 68]	
Proximity to neighbouring houses/housing density	[7, 22, 29, 52, 54]	[7, 22, 29, 52]
*Proximity to periphery of village/cluster*		
Village/cluster location	[4, 16, 42, 63]	[4, 16, 63]
Land cover type/vegetation index/ecological zone	[43, 44, 54]	[54]
Altitude/elevation	[7, 15, 17, 19, 20, 23, 29, 44, 47, 52, 59]	[7, 17, 20, 23, 29, 44, 47, 59]
Slope/aspect	[15, 17, 52]	
Topography (valley shape, wetness index, convergence index)	[23, 52, 54]	[23]
Temperature	[43]	
Rainfall	[15]	
Humidity		
Season	[4, 5, 16, 22, 42, 45, 46, 53]	[4, 16, 22, 42, 45, 46, 53]
*Medical history and genetic factors*		
Previous malaria episodes	[22, 24, 26, 53, 63, 64]	[24, 53]
Duration of residence in malaria-endemic region	[22, 24, 44, 45]	[45]
Antibody titres, incl AMA-1, MSP-2, MSP-1_19	[43, 58]	[43, 58]
Fever history	[21, 64]	[21]
Recent malaria treatment	[25, 26, 28]	[26]
Sickle cell trait	[19, 28]	[28]
G6PD deficiency	[28]	[28]
Hookworm infection	[25]	[25]
Schistosomiasis infection	[26]	[26]
ABO blood group	[27]	[27]
Underweight/BMI	[44, 67]	[44]
Pregnancy status	[60]	
Birth season (for infants and young children)	[19]	[19]
*Plasmodium and human population factors*		
Household malaria cases	[5, 60, 64]	[64]
Local malaria prevalence	[6, 43, 55, 58, 62]	[6, 43, 55, 58]
Malaria prevalence in neighbouring localities	[18, 38]	[18, 38]
Household size/household crowding	[2, 6, 24, 28, 42, 47, 57, 59, 60, 62, 66, 67]	[24, 47, 62]
Village population size/density	[15, 17–19]	[15, 17–19]
*Health seeking behaviour and access to care*		
Level of malaria knowledge	[19, 26]	[26]
Malaria medicine kept at home	[44]	[44]
Distance/access to health facility	[6, 25, 47, 49, 57, 61]	[6, 25, 61]
Access to malaria control program	[7, 38]	[7, 38]
Use of traditional medicine	[38]	

#### Social factors

Most studies (32) included at least one social or behavioural risk factor, mostly individual bed net ownership or use (21 studies) and/or household bed net ownership or use (11 studies) (Table 2). Individual bed net use was associated with a reduced risk of malaria (unadjusted OR 0.63, 95% CI 0.52–0.77, 12 studies; Additional file 1: Figure S1; adjusted OR 0.64, 95% CI 0.54–0.77, nine studies; Additional file 1: Figure S2), however seven studies stated that bed net use was not significant without presenting data. Household bed net ownership was not associated with a reduced risk of malaria (unadjusted OR 0.91, 95% CI 0.66–1.25, six studies; Additional file 1: Figure S3), nor was a household ratio of one to two bed nets per person (unadjusted OR 0.73, 95% CI 0.48–1.09, five studies; Additional file 1: Figure S4). Only two studies reported significant associations for household bed net ownership or ratio and malaria in adjusted models; adjusted estimates were not available for five studies in which unadjusted estimates were presented. A range of other social factors were assessed but replicated in relatively few studies; though there was some evidence that an individual or household member working in an outdoor occupation (four of six studies) and recent travel away from the primary residence (three of six studies) were often associated with increased risk of malaria.

#### Environmental factors

Environmental factors have been extensively studied, especially variables related to housing construction quality and materials, proximity to potential and confirmed breeding sites, proximity to domestic animals and livestock, as well as local landscape features including topography, elevation and land cover (Table 2). House construction characteristics including overall construction quality as well as wall, window, roofing and floor materials were associated with malaria risk in several individual studies (meta-analysis not conducted; see Additional file 1). Malaria risk related to presence and types of eaves (unadjusted OR 1.56, 95% CI 1.18–2.04, four studies; Additional file 1: Figure S5). Other housing features were infrequently associated with malaria risk, including indoor residual spraying (zero of three studies), household water source (one of four studies), solid and liquid waste disposal (zero of four studies) and household surroundings (one of three studies).

Studies measured proximity to vector breeding sites in different ways, including proximity to large water bodies, man-made water storage, forest boundary and agriculture. Increasing distance away from breeding sites was associated with an 11% reduction in malaria risk per 100 m (unadjusted OR 0.89, 95% CI 0.86–0.92, ten studies; Additional file 1: Figure S6). Distance from smaller man-made water storage facilities (including wells, drains, boreholes) was not associated with malaria risk (unadjusted OR 0.99 per 100 m increasing distance, 95% CI 0.95–1.03, six studies; Additional file 1: Figure S7). Proximity to the forest and local forest density were also associated with malaria risk in six of nine studies, all in Asian and Latin American settings (meta-analysis not conducted, see Additional file 1). Topography, elevation and land cover were frequently associated with malaria risk at household (two studies) or cluster level (eight studies, meta-analysis not conducted, see Additional file 1). Variation in malaria risk was consistently observed over altitudinal ranges of 50 m or higher, in both highland and lowland settings. Proximity to agriculture, including irrigated rice fields and plantations, was associated with malaria risk in five of six studies all in African settings. Keeping animals in or near the house was not associated with malaria risk in meta-analysis (unadjusted OR 1.27, 95% CI 0.93–1.73, eight studies; Additional file 1: Figure S8).

Several studies also examined proximity to features of the built environment. Proximity to neighbouring houses, or neighbourhood density, was associated with malaria risk in four of five studies (meta-analysis not done, see Additional file 1). Proximity to a main road was included in three studies but not reported to be significant. Two studies included the number of houses in between a breeding site and the referent participant’s house, one of which reported a significant association. Finally, several studies described “house location” (four studies) or “village/cluster location” (four studies) as exposure variables without further specification, and examined the association with malaria risk. Though three studies reported significant associations with village or cluster location, this was an indicator of, rather than explanatory factor for, observed spatial clustering of malaria. The only study [24] that reported that ‘house location’ was significantly related to malaria risk was conducted in a frontier agricultural settlement, in which house location correlated with duration of residence and proximity to the forest.

#### Medical history and genetic factors

Medical history and genetic factors were less frequently considered than environmental and social factors (Table 2). Previous malaria episodes (two of six studies) as well as duration of residence in a malaria endemic region (one of four studies) were the most frequently studied but with limited association with malaria risk. Positive serology for anti-malaria antibodies strongly predicted malaria risk at individual and cluster level (two of two studies). Hookworm [25] and schistosomiasis [26] infections increased malaria risk in two studies that also show co-infections to be clustered at household level. Genetic traits were studied infrequently and only in African settings but were consistently associated with malaria risk, including ABO blood group [27], sickle cell trait [19] and G6PD deficiency [28].

#### *Plasmodium* and human population factors

Local malaria prevalence was consistently associated with individual-level malaria risk after adjustment for other risk factors, including malaria cases within the household (one of three studies), residence in an identified hotspot (four of five studies based on three datasets), or malaria prevalence in adjoining localities (two of two studies). Of the population-related factors, village population size was associated with increased malaria risk in four of four studies (three studies in similar rural study sites). Household size (four studies) or household crowding (four studies) was associated with increased malaria risk in unadjusted but not adjusted estimates (unadjusted OR for household size 1.08, 95% CI 1.01–1.15; Additional file 1: Figure S9, adjusted estimate presented in one study only; unadjusted OR for household crowding 1.79, 95% CI 1.48–2.16; Additional file 1: Figure S10, adjusted OR 1.12, 95% 0.93–1.35; Additional file 1: Figure S11).

### Health seeking behaviour and access to care

Health seeking behaviour and access to care were infrequently studied (Table 2). Distance to a health facility was associated with malaria risk when using unadjusted but not adjusted study estimates (unadjusted OR 1.59 for ≥1 km from health facility; 95% CI 1.25–2.02, five studies; Additional file 1: Figure S12; reported non-significant after adjustment for other variables in four of five studies). Access to a malaria control program was associated with reduced risk of malaria in the two studies in which it was examined.

### Risk of bias and quality of evidence

The risk of bias and quality of evidence in individual studies varied, with 19 studies considered at low risk of bias (scored eight or nine stars), 24 studies at moderate risk of bias (scored six or seven stars) and eight studies at high risk of bias (scored two to five stars). Variation in bias scores between studies mainly related to adjustment for confounders (14 studies presented only unadjusted estimates, four studies presented minimally adjusted estimates) and lack of description of participation rates. The bias assessment is presented in full in the Additional file 2.

### Variation at individual, household or cluster level

Most studies examined individual and household-level characteristics as risk factors for individual-level malaria infection and separately described aggregated variation in malaria, typically through detection of spatial clusters of malaria based on household of residence. A small number of studies explicitly analysed whether individual-level risk factors for malaria explain variation in risk between villages or other units. In a high-endemicity setting in Ghana [19], there was limited overlap between predictors of individual risk and predictors of village-aggregated risk. Similarly, in Kenya [2] it was found that environmental factors and bed net use poorly predict malaria hotspots, although they do predict individual-level malaria risk. In Tanzania, residence in a hotspot was an independent predictor of malaria risk after adjusting for age, gender, mother’s education, using LLIN, presence of breeding sites, proximity to a health facility and housing quality [6]. In Bangladesh, one study found that spatial variation in malaria could be explained by the same demographic and environmental factors (age, ethnicity, altitude, housing density, forest density) that predict individual-level malaria risk [29], but a subsequent study by the same group that included a broader range of social and environmental variables [7] found that different factors explained individual malaria risk (age, gender, bed net ownership, increased forest cover, elevation and household density) compared to spatial clusters of malaria (ethnicity, forest cover, altitude, floor construction, household density and treatment preference). In a unique approach, a study in Venezuela found that using geographically weighted regression (GWR) models that allow coefficients to vary over space explained a higher proportion of variance than ordinary logistic regression (OLS) [17]. In this study, environmental variables and village population size explained 61–98% of variation for each village in the GWR model. The most significant predictor of individual malaria risk in OLS modelling was the presence of breeding sites within a 1-km radius of the village, but this factor was not significant in every village in the GWR model. Conversely, altitude was identified as a significant risk factor in many villages in the GWR model but was not significant in OLS model.

### Conceptual framework

**Fig. 2:**
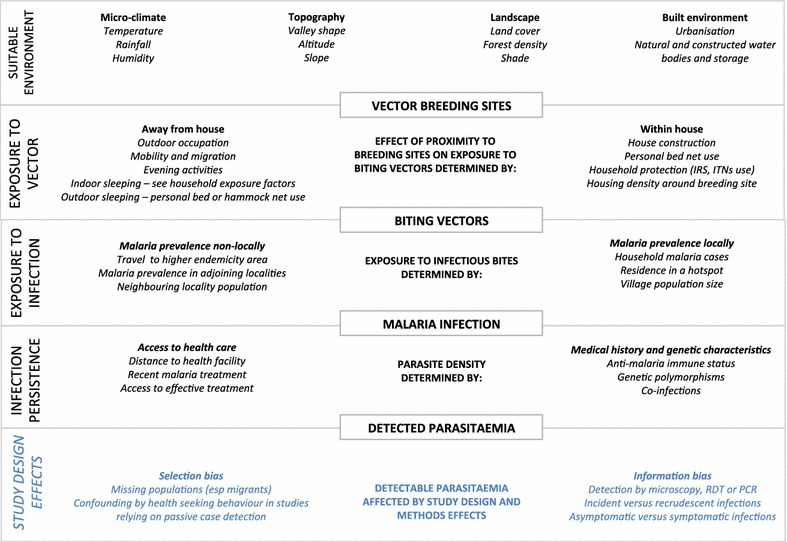
Hierarchical conceptual framework for micro-epidemiology studies of malaria

In this review, several factors that were associated with variation in malaria risk at fine spatial scales were identified. To synthesize the descriptive and quantitative pooled results, a conceptual causal framework for micro-epidemiological studies of malaria is proposed (Fig. 2) that includes all factors consistently associated with malaria risk and highlights how study design may impact findings. The framework is hierarchical, with environmental factors that create the conditions for breeding vector populations at the top of the diagram. Exposure to biting vectors may be influenced by both household-level environmental factors as well as social and behavioural factors, including mobility through landscapes with higher risk of biting vectors, bed net use, outdoor and evening places and activities. Exposure to infectious biting vectors is then determined by local malaria prevalence, or malaria prevalence in travel destinations. Higher malaria prevalence in neighbouring locations may also need to be considered because it may increase the risk of malaria transmission locally. The level of parasitaemia that develops following an infectious bite depends on individual characteristics including immune status (often estimated by duration of residence in a malaria-endemic region), overall health status and co-infections, and genetic traits. Many commonly investigated risk factors are not represented, such as education, wealth status or malaria-related knowledge, because there was no consistent evidence that these variables are associated with malaria risk at micro-epidemiological scales in the studies in this review. Furthermore, from a causal perspective, these variables have indirect effects on malaria that should manifest in exposure-related factors that more directly influence malaria risk.

## Discussion

This review presents the first attempt to systematically identify risk factors that explain local variation in malaria risk. Several risk factors were identified that are consistently associated with malaria risk at fine spatial scales, including individual bed net use, presence of open eaves in housing construction, proximity to vector breeding sites, household size and crowding, and distance to a health facility. In the studies screened and included in this review, no clear description of what micro-epidemiology should entail was found. It is proposed that micro-epidemiology should aim to explain local variation, where ‘local’ implies a transmission network (or a component of it) that is characteristic of a defined socio-spatial aggregation (such as a village) and ‘variation’ describes heterogeneity in malaria risk between *groups* of individuals, clustered in socio-spatial aggregations such as households, sub-village clusters, villages or urban zones. Although several studies linked the purpose of micro-epidemiological analysis to more efficient identification and targeting of malaria hotspots, this review demonstrates that there is limited evidence that variation in malaria risk at household, sub-village cluster or higher-level units can be fully explained by individual-level risk factors. Therefore, explaining local variation requires that analyses at the level of individual-level risk factors must be related to higher-level units at which heterogeneity in malaria risk occurs, including analysis of how risk factors interact or reinforce each other in the local context to potentiate malaria transmission.

Through this review, several issues were identified that should be considered when planning micro-epidemiological studies, including challenges associated with small sample sizes, units of analysis, and interdisciplinary approaches.

### Sample size of micro-epidemiology studies

The minimum number of villages or other socio-spatial aggregations to include in micro-epidemiological studies remains unclear. It is proposed that the number of higher-level units that are included should reflect the underlying transmission network; that is, if variation within a village is hypothesized to relate to variation in malaria risk between contiguous villages in the area, then sufficient units should be included to allow between-village differences to be explored. One study [18] showed how malaria risk spreads from larger to smaller villages in spatially contiguous localities but no other study explicitly considered factors that explain transmission linkages between villages. As mobile and migrant people have been shown to be at higher risk of malaria in diverse settings [30], local mobility patterns should be explored to explain local malaria risk.

Additionally, small sample sizes may limit statistical power to detect important risk factors, and effect sizes may be consistently over-estimated [31], which can bias meta-analyses. Overcoming these challenges in part requires reduced reliance on statistical testing alone for assessing which risk factors are important, which can lead to misleading and spurious results [32].

### Analysis of aggregated malaria risk

The studies included in this review analysed aggregated malaria risk mostly through spatial analyses, in which the registered domicile address is taken as the primary spatial unit for assessing spatial clustering and measuring household-based risk factors for malaria infection. This approach implicitly assumes that malaria transmission is occurring in the vicinity of the village-based household. However in several settings, especially South-East Asia and Latin America, occupation-related mobility and multiple residence systems are associated with malaria risk [33–35]. In an urban east African setting, scattered distribution of malaria infections with transient hotspots that do not correlate with vector population density has been described [36], but there is little information on how this epidemiological pattern arises. This implies that the primary unit of spatial analysis should be the risk locations where people spend time during vector-biting hours, rather than only the registered domicile address.

### Confounding and study design

An interdisciplinary research design is integral to micro-epidemiology, as the lack of inclusion of data from different disciplines contributes to unmeasured confounding. For example, estimates of the effect of proximity to breeding sites in individual studies, the most frequently studied risk factor for malaria infection, were in some cases substantially attenuated after adjustment [37], or not at all [2], and it remains unclear whether the effect of proximity to a breeding site is modified or confounded by housing structure, mobility patterns, individual protective measures, and other factors. In general, demographic, social, population and other risk factors may confound studies limited to environmental factors, but many studies do not include these variables, which limits the evidence base on which control programmes can assess which risk factors could be targets for intervention in their setting. Of further note is that genetic traits and co-infections with non-*Plasmodium* pathogens were only considered in African study settings. As these factors were consistently associated with malaria infection, micro-epidemiological malaria studies in other settings should consider including more genetic and clinical characteristics, as these characteristics may explain some heterogeneity in malaria infection that has different implications for intervention strategies. The role of health services and health systems was rarely considered; only two studies explicitly measured variation in access to malaria control programs, but no studies considered the effectiveness and acceptability of malaria control and other health care problems as a source of micro-epidemiological variation in malaria risk. Similarly, there is scope for more detailed research on how specific local socioeconomic conditions modify malaria risk, and the pathways through which this occurs, which goes beyond simple descriptions of individual or household-level socioeconomic status.

Only one study [38] used a mixed-methods design to contextualize risk factors to explain local malaria epidemiology. When well conducted, qualitative methodologies can be used to enrich and contrast quantitative data and lead to insights about how risk factors intersect and reinforce each other to promote or hinder malaria transmission. Mixed methods designs offer an alternative paradigm for describing the validity and transferability of study findings, which may be more robust than statistical and quantitative inference alone [39].

### Bias and limitations

Several sources of bias limit the strength of the evidence on risk factors underlying micro-epidemiological patterns in malaria risk across different settings. As there has been no consistent use of the term ‘micro-epidemiology’ or other terms to describe studies that analyse variation in malaria risk within or between sub-village clusters or villages, defining a search strategy was not straightforward, and some potentially relevant studies may have been missed. Future reviews on this topic could consider a broader use of keywords. The choice of malaria diagnostic (microscopy, RDT or PCR) may impact the observed variation in malaria risk, as risk factors for asymptomatic carriage may differ from those for clinical cases particularly in high-endemicity settings. Passive case detection, compared to active case detection or population screening, may introduce confounding by health seeking behavior and miss groups of individuals at higher risk but with less access to care.

Across studies, there was a substantial risk of bias, given that studies frequently excluded effect estimates for variables with reportedly non-significant associations with malaria risk. Most meta-analysis estimates presented are calculated using unadjusted findings. Furthermore, there were insufficient studies to conduct meta-analyses stratified on different *Plasmodium* species, vectors, at-risk populations and study design, but this limits the utility of the meta-analysis results. For example, keeping animals in or near the house was not associated with malaria risk overall, but as this varies substantially with vector and host species, as well as extent of urbanization, this risk factor may be important in specific settings. In this review, studies that measured serological as well as parasitological outcomes were pooled, as there is evidence that seroprevalence of anti-malarial antibodies may be a more stable marker of recent malaria risk at micro-epidemiological scales [2]. However, serological outcomes may reflect both recent as well as past exposure, which may obscure risk factors for recent infection in these studies. Due to the small number of studies in most meta-analyses, it was not feasible to conduct separate meta-analyses for studies reporting only serological outcomes, but this should be considered for future meta-analyses.

## Conclusion

Conceptual recognition of the relevance of micro-epidemiology for malaria control is not new; as expressed by Hackett in 1937, “everything about malaria is so molded by local conditions that it becomes a thousand epidemiological puzzles” [40]. However there has been limited attention towards developing theory for micro-epidemiology, encompassing a practical definition and methods. As malaria-endemic countries aim to reach elimination goals, there will be increasing need to target persistent and highly heterogenous malaria transmission at small spatial scales using differential interventions that reflect local transmission characteristics. To achieve this, methods that recognize and engage with sources of local variation whilst achieving a level of transferability of research findings between settings, and from research to practice, are required. Exploring risk factors *in context* rather than comparing isolated risk factors for individual-level infection would allow us to understand how different risk factors combine to produce variation in malaria risk at aggregated rather than just individual level. The conceptual framework proposed in this review attempts to identify and structure relevant risk factors that were frequently associated with malaria risk in micro-epidemiological studies, which will contribute to progress in theorization and assist in planning of future studies. Further research is required to fully operationalise the concept of micro-epidemiology and incorporate it into discussions of malaria elimination strategies.

## Additional files

**Additional file 1.** Meta-analysis forest plots. This file includes detailed methods and forest plots for all meta-analyses conducted, as well as additional information on why meta-analysis could not be conducted for some risk factors.

**Additional file 2.** Risk of bias in studies included in systematic review. This table provides a detailed bias assessment and score across the three domains of the Newcastle–Ottawa Quality Assessment Scale.
