# Supplementary material for: Defining micro-epidemiology for malaria elimination: systematic review and meta-analysis
Source: Malar J. 2017 Apr 20;16:164. doi: 10.1186/s12936-017-1792-1 (PMC5399382; doi:10.1186/s12936-017-1792-1)
Supplement: Supplementary file 1 — Additional file 1. Meta-analysis forest plots. This file includes detailed methods and forest plots for all meta-analyses conducted, as well as additional information on why meta-analysis could not be conducted for some risk factors. [file 12936_2017_1792_MOESM1_ESM.docx]

**Meta-analysis methods and results**

Methods

Results from individual studies were combined in meta-analyses to estimate the magnitude of effect sizes and heterogeneity of effects across studies. Several methods were used to generate effect estimates that were not presented in the required form for meta-analysis in the individual publications. Firstly, estimates were calculated directly from frequency tables if available. Secondly, the inverse of estimates with two exposure levels was taken if required to obtain a common referent level for odds ratio measures. For exposures with three or more levels, the method of Hamling[1] was used to recalculate the exposure for the required referent level. For variables describing proximity to a breeding site, in which distance was presented as a categorical variable, the method of Greenland and Longnecker[2] was used to estimate a linear dose-response association and express the unit change in malaria risk per 100m increasing distance from the breeding site, taking correlation of the effect estimates with the reference category into account. Exposure scores were assigned as the mid-point of the category range, or for unbounded upper categories, as the upper bound of the second-last interval plus two times the width of the second-to-last interval, based on sensitivity analysis of one study for which the distribution of the distance exposures was known[3]. The Greenland Longnecker method was implemented using the *glst* command in Stata[4]. For studies that treated proximity to a breeding site as a continuous linear variable, effect estimates were re-scaled to express the change in malaria odds per 100m increase in distance from the breeding site.

Meta-analysis was conducted if there were at least 4 studies per variable with comparable exposures and unadjusted and/or adjusted estimates of the effect and 95% confidence intervals were presented in the article or could be calculated as described above. For each variable, unadjusted estimates were pooled, and if available, adjusted estimates were pooled separately, without further stratification on variables were adjusted for in each study due to the small number of studies in each meta-analysis. We chose a minimum of 4 studies per variable for meta-analysis as we assumed *a priori* that effects would vary between studies due to different underlying at-risk populations, vectors, *Plasmodium* species and study designs, and therefore selected to use random effects models to calculate pooled estimates. Secondly, we aimed to qualitatively describe heterogeneity in effect sizes in different studies, and overall considered that meta-analyses based on 2 or 3 studies would be largely uninformative given these considerations. Meta-analyses were not conducted for demographic variables as variables such as sex, ethnicity, and migrant status which were considered *a priori* as more distal risk factors that affect malaria risk through their various effects on living conditions, environmental and occupational exposures and health-related behaviours. Though acquired anti-malarial immunity is age-dependent, we did not conduct a meta-analysis for age because without seroconversion data for most studies, the unique effect of age cannot be distinguished from the effect of age on exposure-related behaviours.

Meta-analyses were implemented using *metan* command with random effects and inverse variance options in Stata/IC 13. Pooled estimates are presented in-text where calculated, otherwise the total number of studies assessing each variable and the number of significant associations reported are described. Heterogeneity by relative risk measure (odds ratio, rate ratio, risk ratio) was explored qualitatively but there were too few studies per variable to stratify on study design or risk measure, therefore all effect estimates were assumed to estimate the odds ratio, as this was the most commonly calculated measure. Heterogeneity between study estimates included in meta-analysis was assessed using the I^2^ statistic.

References for this section

1. Hamling, J., et al., *Facilitating meta-analyses by deriving relative effect and precision estimates for alternative comparisons from a set of estimates presented by exposure level or disease category.* Statistics in Medicine, 2008. **27**(7): p. 954-970.

2. Greenland, S. and M.P. Longnecker, *Methods for Trend Estimation from Summarized Dose-Response Data, with Applications to Meta-Analysis.* American Journal of Epidemiology, 1992. **135**(11): p. 1301-1309.

3. Brooker, S., et al., *Spatial clustering of malaria and associated risk factors during an epidemic in a highland area of western Kenya.* Trop Med Int Health, 2004. **9**(7): p. 757-66.

4. Orsini, N., R. Bellocco, and S. Greenland, *Generalized least squares for trend estimation of summarized dose-response data.* Stata Journal, 2006. **6**(1): p. 40.

Results – Forest Plots

**1. Social factors**

- 1. **Bed net ownership and use**

**Figure S1: Meta-analysis of the association between self-reported individual bed net use and malaria infection using unadjusted study estimates**

**Unadjusted odds ratio for bed net use compared to no use**

*Bed net use increases odds of malaria*

*Bed net use decreases odds of malaria*

**Figure S2: Meta-analysis of the association between self-reported individual bed net use and malaria infection using adjusted study estimates**

**Adjusted odds ratio for bed net use compared to no use**

*Bed net use increases odds of malaria*

*Bed net use decreases odds of malaria*

**Figure S3: Meta-analysis of the association between household bed net ownership and individual malaria infection**

**Unadjusted odds ratio for household owning 1 or more bed net compared to no net**

*Household bed net ownership increases odds of malaria*

*Household bed net ownership decreases odds of malaria*

**Figure S4: Meta-analysis of the association between high versus low household bed net ownership ratio and individual malaria infection**

**Unadjusted odds ratio for household bed net ratio of 1-2 people per net versus more than 2 people per net**

*More bed nets per person increases odds of malaria*

*More bed nets per person decreases odds of malaria*

1. **Environmental factors**
   1. **Housing characteristics**

**Figure S5: Meta-analysis of the association between presence of open eaves in housing and individual malaria infection**

**Unadjusted odds ratio for open eaves compared to closed eaves**

*Open eaves increase odds of malaria*

*Open eaves decrease odds of malaria*

- 1. **Proximity to breeding sites and water bodies**

**Figure S6: Meta-analysis of the association between household distance to breeding site and individual malaria infection**

**Unadjusted odds ratio per 100m increase**

*Increasing distance from a breeding site increases odds of malaria*

*Increasing distance from a breeding site*

*decreases odds of malaria*

**Figure S7: Meta-analysis of the association between household distance to man-made water storage units and individual malaria infection**

**Unadjusted odds ratio per 100m increase**

*Increasing distance from a small water storage unit increases odds of malaria*

*Increasing distance from a small water storage unit decreases odds of malaria*

- 1. **Proximity to alternative hosts**

**Figure S8: Meta-analysis of the association between animals kept in or near the house and individual malaria infection**

**Unadjusted odds ratio for animals kept in or near the house compared to animals not kept near house**

*Animals kept in or near the house increases odds of malaria*

*Animals kept in or near the house*

*decreases odds of malaria*

1. **Plasmodium and human population**

**3.1. Household size**

**Figure S9: Meta-analysis of the association between household size and individual malaria infection**

**Unadjusted odds ratio per additional household member**

*Larger household size increases odds of malaria*

*Larger household size decreases odds of malaria*

**3.2. Household crowding**

**Figure S10: Meta-analysis of the association between household crowding and individual malaria infection using unadjusted study estimates**

**Unadjusted odds ratio for 5 or more household members compared to less than 5 household members**

*Household crowding increases odds of malaria*

*Household crowding decreases odds of malaria*

**Figure S11: Meta-analysis of the association between household crowding and individual malaria infection using adjusted study estimates**

**Adjusted odds ratio for 5 or more household members compared to less than 5 household members**

*Household crowding increases odds of malaria*

*Household crowding decreases odds of malaria*

1. **Health seeking behavior and access to care**

**Figure S12: Meta-analysis of the association between distance to a health facility and individual malaria infection**

**Unadjusted odds ratio for distance to a health facility greater than 1km compared to less than 1km**

*Increased distance from health centre increases odds of malaria*

*Increased distance from health centre decreases odds of malaria*

**Reasons why variables were excluded from meta-analysis**

**Social factors**

- Recent travel: not comparable exposures – travel to rural areas, any travel, nights spent sleeping away from camp
- Outdoor occupation: Estimates available for one study only

**Environmental factors**

- Housing construction overall: Not presented (Clark, Pullan B) or with insufficient data for meta-analysis (Gamage); available for 3 studies (Mosha, van der hoek, Gunawardena, all compared poor vs good).
- House roof: Reference categories not specified in 2/7 studies, results not presented in 2/7 studies, leaving only 3 studies
- House floor: Estimates not presented for 2 studies, estimates available for 3 studies
- Previous malaria episodes: Different exposure categories. Including since birth, since the past rainy season, in the past *n* months.
- Presence/type of windows: Not presented (da Silva, Bousema), exposure variables not directly comparable (presence of windows, presence of screened windows, number of windows, window are) and outcome not directly comparable (individual infection versus residence in hotspot).
- Proximity to agriculture: data presented in 5 of 6 studies, but exposures not directly comparable (use of irrigated land, distance to rice paddies <750m, farm within 30m, effect of construction of large dam as part of a rice irrigation project)
- Elevation: different ranges, some lowland, some highland settings, not directly comparable.
- Neighbourhood housing density: 5 studies based on 4 datasets, Exposures not readily comparable except for 3 Haque et al studies from same/similar site

**Epidemiological factors**

- Residence in a hotspot: lack of consistency in how hotspots were defined, different outcomes (PCR, serology), 5 studies based on 3 datasets
