# Supplementary material for: Defining micro-epidemiology for malaria elimination: systematic review and meta-analysis
Source: Malar J. 2017 Apr 20;16:164. doi: 10.1186/s12936-017-1792-1 (PMC5399382; doi:10.1186/s12936-017-1792-1)
Supplement: Supplementary file 2 — Additional file 2. Risk of bias in studies included in systematic review. This table provides a detailed bias assessment and score across the three domains of the Newcastle–Ottawa Quality Assessment Scale. [file 12936_2017_1792_MOESM2_ESM.pdf]

The Newcastle-Ottawa Scale for assessing the quality of nonrandomised studies in meta-analyses

Case control studies

		Selection				Comparability	Exposure			Star total
**Author**	**Year**	**Case definition adequate**	**Representativeness of cases**	**Selection of controls**	**Definition of controls**	**Controls for risk factors from one (★) or two (★★) groups in classification scheme**	**Ascertainment**	**Same ascertainment for cases and controls**	**Non-response non differential**	
Alemu, Worku et al	2014	Yes, with independent validation (by microscopy or RDT) ★	Consecutive series ★	Community controls ★	No malaria infection (by microscopy or RDT) ★	Adjusted for age, sex, travel ★	Interview not blinded to case/control status	Yes ★	Not described	6
Brooker, Clarke et al	2004	Yes, with independent validation (by microscopy) ★	Consecutive series (school children) ★	Community controls ★	No malaria symptoms or negative by microscopy ★	Matched on age and school, adjusted for underweight, medicine at home, altitude ★★	Interview not blinded to case/control status	Yes ★	Not described	7
Rosas-Aguirre, Ponce et al	2015	Yes, through record linkage ★	Representative sampling ★	Community controls ★	No passively detected malaria case in household (does not exclude malaria infection)	Adjusted for distance to drain, household size, potable water availability, animal ownership ★	Interview not blinded to case/control status	Yes ★	Not described	5

Cross sectional studies

		Selection				Comparability	Exposure			Star total
**Author**	**Year**	**Case definition adequate**	**Representativeness of cases**	**Selection of controls**	**Definition of controls**	**Controls for risk factors from one (★) or two (★★) groups in classification scheme**	**Ascertainment**	**Same ascertainment for cases and controls**	**Non-response non differential**	
Atieli, Zhou et al	2011	Yes, with independent validation (by microscopy) ★	Representative random sampling ★	Community controls ★	No malaria infection (by microscopy) ★	No adjustment for confounders	Structured interview blind to infection status ★	Yes ★	Not described	6
Audibert, Josseran et al	1990	Yes, with independent validation (by microscopy) ★	Representative random sampling ★	Community controls ★	No malaria infection (by microscopy) ★	No adjustment	Structured interview blind to infection status ★	Yes ★	Not described	6
Baragatti, Fournet et al	2009	Yes, with independent validation (by microscopy) ★	Representative random sampling ★	Community controls ★	No malaria infection (by microscopy) ★	Controls for age, bed net use, equipment and education levels, season, land tenure, distance to drinking fountain ★★	Structured interview blind to infection status ★	Yes ★	Not described	8
Cattani, Moir et al	1986	Yes, with independent validation (by microscopy) ★	Census sampling ★	Community controls ★	No malaria infection (by microscopy) ★	No adjustment	Structured interview blind to infection status ★	Yes ★	Participation rate exceeded 78% overall ★	7
Florey, King et al	2012	Yes, with independent validation (by PCR) ★	Census sampling ★	Community controls ★	No malaria infection (by PCR) ★	Controls for age, recent malaria treatment, malaria knowledge, household socioeconomic status, outdoor night activity ★★	Structured interview blind to infection status ★	Yes ★	Participation rate 41.3%, adult males under-represented	8
Haque, Magalhaes et al	2011	Yes, with independent validation (by RDT) ★	Representative random sampling ★	Community controls ★	No malaria infection (by RDT) ★	Controls for age, ethnicity, altitude, forest density, household density ★★	Structured interview blind to infection status ★	Yes ★	Not described	8

Cross sectional studies continued

Haque, Sunahara et al	2011	Yes, with independent validation (by RDT) ★	Representative random sampling ★	Community controls ★	No malaria infection (by RDT) ★	Controls for ethnicity, occupation, bed net ownership, forest density, altitude, floor materials, household density, access to malaria control program ★★	Structured interview blind to infection status ★	Yes ★	Not described	8
Mosha, Sturrock et al	2014	Yes, with independent validation (by PCR) ★	Representative random sampling ★	Community controls ★	No malaria infection (by PCR) ★	Controls for age, sex, bed net use, household quality, education, proximity to a breeding site, distance to a health facility, residence in a hotspot ★★	Structured interview blind to infection status ★	Yes ★	Not described	8
Mosha, Sturrock et al	2013	Yes, with independent validation (by PCR) ★	Census sampling ★	Community controls ★	No malaria infection (by PCR) ★	Controls for age and household exposure ★	Structured interview blind to infection status ★	Yes ★	Not described	7
Murhandarwati, Fuad et al	2014	Yes, with independent validation (by microscopy) ★	All cases selected ★	No controls	No controls	No adjustment, descriptive and qualitative only	Structured interview not blinded to infection status	N/A	Not described	2
Ndiath, Faye et al	2014	Yes, with independent validation (by microscopy and RDT) ★	Representative random sampling ★	Community controls ★	No malaria infection (by microscopy) ★	Controls for age, materials of sleeping rooms, materials of floor, window in room, distance to livestock, bed net use, goat ownership, socioeconomic status of household ★ ★	Structured interview blind to infection status ★	Yes ★	Not described	8
Nixon, Nixon et al	2014	Yes, with independent validation (by microscopy) ★	Representative random sampling ★	Community controls ★	No malaria infection (by microscopy) ★	Controls for age, distance to breeding sites, distance to health facility, number of household residents ★ ★	Structured interview blind to infection status ★	Yes ★	Not described	8

Cross sectional studies continued

Prakash, Mohapatra	2000	Yes, with independent validation (by microscopy) ★	Census sampling ★	Community controls ★	No malaria infection (by microscopy) ★	No adjustment for confounders	Structured interview blind to infection status ★	Yes ★	Not described	6
Pullan, Bukirwa et al	2010	Yes, with independent validation (by microscopy and RDT) ★	Census sampling ★	Community controls ★	No malaria infection (by microscopy) ★	Controls for age, distance to agriculture, distance to health centre ★★	Structured interview blind to infection status ★	Yes ★	Not described	8
Pullan, Kabatereine et al	2011	Yes, with independent validation (by microscopy and RDT) ★	Census sampling ★	Community controls ★	No malaria infection (by microscopy) ★	Controls for age, bed net use, distance to agriculture, distance to health centre, hookworm infection ★★	Structured interview blind to infection status ★	Yes ★	Not described	8
Rosas-Aguirre, Speybroeck et al	2015	Yes, with independent validation (by PCR) ★	Representative random sampling ★	Community controls ★	No malaria infection (by PCR) ★	Controls for age, location, outdoor occupation ★★	Structured interview blind to infection status ★	Yes ★	Not described	8
Rulisa, Kateera et al	2013	Yes, with independent validation (by microscopy and RDT) ★	Potential for selection bias (health centre recruitment)	Health centre controls	No malaria infection (by microscopy) ★	Controls for age, sex, household member with malaria, bed net ownership, house wall material, house roof material, open water vessel in household, environment around household, household electricity ★★	Structured interview blind to infection status ★	Yes ★	Not described	6
Sissoko, van den Hougen et al	2015	Yes, with independent validation (by microscopy) ★	Potential for selection bias (random sampling repeated until minimum number of consenting individuals participated)	Community controls ★	No malaria infection (by microscopy) ★	Controls for bed net use, open eaves, window surface and other confounders not further described ★★	Structured interview blind to infection status ★	Yes ★	Participation rate exceeded 90% of consenting individuals, proportion of non-consenting individuals unknown	7

Cross sectional studies continued

Trape, Lefebvre-Zante et al	1992	Yes, with independent validation (by microscopy) ★	Representative random sampling ★	Community controls ★	No malaria infection (by microscopy) ★	No adjustment	Structured interview blind to infection status ★	Yes ★	Not described	6
Winskill, Rowland et al	2011	Yes, with independent validation (by microscopy) ★	Census sampling ★	Community controls ★	No malaria infection (by microscopy) ★	Controls for age, sex, bed net use ★★	Structured interview blind to infection status ★	Yes ★	Not described	8

Cohort studies

		Selection				Comparability	Outcome			Star total
**Author**	**Year**	**Representativeness of exposed**	**Representativeness of non-exposed**	**Exposure ascertainment**	**Outcome not present at start**	**Controls for risk factors from one (★) or two (★★) groups in classification scheme**	**Ascertainment**	**Follow up sufficient**	**Completion rate**	
Barrera, Grillet et al	1999	Somewhat representative of the inhabitants of the study area ★	Drawn from same community as exposed cohort ★	Structured interview ★	No	Adjusts for number of inhabitants, density of breeding sites, distance to breeding sites ★★	Record linkage ✯	Yes (12 years) ✯	Not described	7
Barros and Honario	2015	Truly representative sampling of children in the study area ★	Drawn from same community as exposed cohort ★	Structured interview ★	No	No adjustment	Independent blind assessment (by microscopy) ✯	Yes (2.5 years) ✯	Not described	5
Basurko, Demattei et al	2013	Selected group (permanent residents only, despite high proportion of local population being migrants)	Drawn from same community as exposed cohort ★	Structured interview ★	No	No adjustment	Independent blind assessment (by microscopy) ✯	Yes (6 years) ✯	Not sufficiently described to assess	4
Bejon, Williams et al	2010	Truly representative sampling of children in the study area ★	Drawn from same community as exposed cohort ★	Structured interview ★	No	Adjusts for vegetation index, day time temperature, night time temperature, middle infra-red bi-annual phase, parasite genotyping (MSP2), antibody titres ✯✯	Independent blind assessment (by microscopy and PCR) ✯	Yes (12 years) ✯	Not described	7
Bousema, Drakeley et al	2010	Selected group (clinical trial participants)	Drawn from same community as exposed cohort ★	Structured interview ★	No	Adjusts for distance to breeding site, wealth index, roof material, wall material, wall structure, bed net use ✯✯	Independent blind assessment (by RDT) ✯	Yes (4 years) ✯	Not described	6

Cohort studies continued

Camargo, Ferreira et al	1994	Truly representative sampling of the inhabitants in the study area ★	Drawn from same community as exposed cohort ★	Structured interview ★	No	No adjustment	Independent blind assessment (by microscopy) ★	Yes (1 year) ★	Follow up rate <80% due to high population mobility, but open cohort well documented and accounted for at each survey point ★	6
Clark, Greenhouse et al	2008	Truly representative sampling of children in the study area ★	Drawn from same community as exposed cohort ★	Structured interview ★	Yes ★	Adjusts for age, sex, sickle cell trait, G6PD status, bed net use, wealth index, household crowding, distance from swamp ★★	Independent blind assessment (by microscopy and PCR) ★	Yes (2 years) ★	Loss to follow-up unlikely to cause bias (>80% follow up) ★	9
Coulibaly, Rebaudet et al	2013	Truly representative sampling of children in the study area ★	Drawn from same community as exposed cohort ★	Structured interview ★	No	No adjustment	Independent blind assessment (by microscopy and PCR) ★	Yes (5 years) ★	Not described	5
da Silva-Nunes, Codeco et al	2008	Truly representative sampling of the inhabitants in the study village ★	Drawn from same community as exposed cohort ★	Structured interview ★	No	Adjusts for gender, main occupation, years of residence in the area, recreational fishing, house location, number of inhabitants in the house, wealth index, sharing house with a land cleare❒ ★★	Independent blind assessment (by microscopy and PCR) ★	Yes (14 months) ★	Not described	7
de Barros, Honario and Arruda	2011	Somewhat representative of the inhabitants of the study area ★	Drawn from same community as exposed cohort ★	Structured interview ★	No	Adjusts for distance to breeding sites, distance to water collections, number of nights slept out of the house ★★	Record linkage ★	Yes (2.5 years) ★	Not described	7
Ernst, Adoka et al	2006	Truly representative sampling of the inhabitants in the study village ★	Drawn from same community as exposed cohort ★	Structured interview ★	No	Adjusts for metal roof, number in household, distance to swamp, distance to forest edge, elevation ★★	Record linkage ★	Yes (4 years) ★	Not described	7

Cohort studies continued

Gamage-Mendis, Carter et al	1991	Truly representative sampling of the inhabitants in the study village ★	Drawn from same community as exposed cohort ★	Structured interview ★	No	No adjustment	Independent blind assessment (by microscopy) ★	Yes (17 months) ★	Not described	5
Gaudart, Poudiougou et al	2006	Truly representative sampling of children in the study village ★	Drawn from same community as exposed cohort ★	Structured interview ★	Yes ★	Adjusts for age, access to treatment, thatched roofs, seasonality, presence of breeding sites ★★	Independent blind assessment (by microscopy) ★	Yes (5 years) ★	Loss to follow-up unlikely to cause bias (>80% follow up) ★	9
Ghebreyesus, Haile et al	2000	Truly representative sampling of children in the study village ★	Drawn from same community as exposed cohort ★	Structured interview ★	No	Adjusts for age, sex, roof material, type of eaves, windows, number of sleeping rooms, separate kitchen, animals sleep in house, number of animals owned, radio owned, source of water in dry season, source of water in wet season, use of irrigated land ★★	Independent blind assessment (by microscopy) ★	Yes (12 months) ★	Loss to follow-up unlikely to cause bias (>90% follow up) ★	8
Grange, Loucoubar et al	2015	Truly representative sampling of the inhabitants of the study village ★	Drawn from same community as exposed cohort ★	Structured interview ★	No	Adjusts for age, type of anti-malarial drug used in study area at time, recent malaria episode, ABO blood group ★ ★	Independent blind assessment (by microscopy) ★	Yes (19 years) ★	Not described	7
Grillet, Barrera et al	2010	Somewhat representative of the inhabitants of the study area ★	Drawn from same community as exposed cohort ★	Structured interview ★	Yes ★	Adjusts for terrain elevation, terrain slope, number of inhabitants per village, distance to the main road, breeding site density, distance to breeding site, distance to wetland ★★	Record linkage ★	Yes (7 years) ★	Not described	8
Grillet, Jordan, Fortin	2010	Somewhat representative of the inhabitants of the study area ★	Drawn from same community as exposed cohort ★	Structured interview ★	No	No adjustment	Record linkage ★	Yes (7 years) ★	Not described	5

Cohort studies continued

Gunawardena, Wickremasinghe et al	1998	Truly representative sampling of the inhabitants of the study villages ★	Drawn from same community as exposed cohort ★	Structured interview ★	No	No adjustment	Record linkage ★	Yes (18 months) ★	Not described	5
Haque, Glass et al	2013	Somewhat representative of the inhabitants of the study area ★	Drawn from same community as exposed cohort ★	Structured interview ★	No	Adjusts for bed net ratio, wall material, housing density, proximity to streams, aspect, elevation, convergence index, wetness index ★ ★	Record linkage ★	Yes (2 years) ★	Not described	7
Kreuels, Kobbe et al	2008	Selected population (children enrolled in randomised controlled trial)	Drawn from same community as exposed cohort ★	Structured interview ★	No	Controls for sex, ethnicity, birth season, sickle cell trait, mother's education, mother's occupation, knowledge of malaria, bed net use, screened windows, household socieconomic status ★★	Independent blind assessment (by microscopy) ★	Yes (2 years) ★	Not described	6
Loha, Lunde et al	2012	Truly representative sampling of the inhabitants of the study village ★	Drawn from same community as exposed cohort ★	Structured interview ★	No	Controls for age, sex, wealth index, total nights spent under ITN, distance from breeding site, household density ★★	Independent blind assessment (by microscopy) ★	Yes (2 years) ★	Not described	7
Luxemburger, Thwai et al	1996	Somewhat representative of the residents of the study area ★	Drawn from same community as exposed cohort ★	Structured interview ★	Yes ★	No adjustment	Independent blind assessment (by microscopy) ★	Yes (12 months) ★	Loss to follow-up unlikely to cause bias (>80% completion)	7
Midega, Smith et al	2012	Truly representative sampling of children in the study villages ★	Drawn from same community as exposed cohort ★	Structured interview ★	No	Controls for housing density, population density, gradient of slope, wind direction, number of larvae ★★	Independent blind assessment (by microscopy) ★	Yes (12 months) ★	Loss to follow-up unlikely to cause bias (>80% follow up) ★	8

Cohort studies continued

Olotu, Fegan et al	2012	Truly representative sampling of children in the study villages ★	Drawn from same community as exposed cohort ★	Structured interview ★	No	Controls for residence in a hotspot, age ✯	Independent blind assessment (by microscopy) ✯	Yes (12 years) ✯	Not sufficiently described to assess	6
Parker, Matthews et al	2015	Truly representative sampling of the inhabitants of the study village ★	Drawn from same community as exposed cohort ★	Structured interview ★	No	Controls for age, sex, citizenship, migrant status, elevation, dependency ratio ✯✯	Independent blind assessment (by microscopy) ✯	Yes (10 months) ✯	Loss to follow-up unlikely to cause bias (>80% follow up) ✯	8
Peterson, Borrell et al	2009	Somewhat representative of the residents of the study area ★	Drawn from same community as exposed cohort ★	Structured interview ★	No	Controls for age, sex, marital status, indoor work, regular/recent travel, distance to breeding sites, tidy compound, household preventive measures, number of livestock animals, number of children in household ✯✯	Independent blind assessment (by microscopy) ✯	Yes (4 months) ✯	Not sufficiently described to assess	7
van der Hoek, Konradsen et al	1998	Truly representative sampling of individuals in the study village ★	Drawn from same community as exposed cohort ★	Structured interview ★	Yes ★	Controls for age, gender, household size, socioeconomic status, housing quality, distance to cattle shed, distance to stream, use of bed net, use of pyrethrum coils, use of traditional fumigants ✯✯	Independent blind assessment (by microscopy) ✯	Yes (12 months) ★	Not sufficiently described to assess	8
Ye, Kyobutungi et al	2007	Truly representative sampling of children in the study villages ★	Drawn from same community as exposed cohort ★	Structured interview ★	No	Controls for age, gender, ethnicity, location, net use, proximity to well, proximity to farm, proximity to animal enclosure, proximity to breeding site, season ✯✯	Independent blind assessment (by microscopy) ✯	Yes (12 months) ★	Loss to follow-up unlikely to cause bias (>80% follow up) ✯	8
